# Synthesis and Characterization of GO/ZIF-67 Nanocomposite: Investigation of Catalytic Activity for the Determination of Epinine in the Presence of Dobutamine

**DOI:** 10.3390/mi13010088

**Published:** 2022-01-06

**Authors:** Mahboobeh Shahsavari, Mojtaba Mortazavi, Somayeh Tajik, Iran Sheikhshoaie, Hadi Beitollahi

**Affiliations:** 1 Department of Chemistry, Faculty of Science, Shahid Bahonar University of Kerman, Kerman 7616914111, Iran; mahboob92sh@gmail.com (M.S.); i_shoaie@yahoo.com (I.S.); 2 Biotechnology Department, Institute of Science and High Technology and Environmental Sciences, Graduate University of Advanced Technology, Kerman 7631885356, Iran; mortezavimm@gmail.com; 3 Research Center of Tropical and Infectious Diseases, Kerman University of Medical Sciences, Kerman 7616913555, Iran; 4 Environment Department, Institute of Science and High Technology and Environmental Sciences, Graduate University of Advanced Technology, Kerman 7631885356, Iran

**Keywords:** epinine, dobutamine, voltammetric sensors, screen printed electrode, GO/ZIF-67 nanocomposite

## Abstract

In this study, we prepared graphene oxide (GO)/ZIF-67 nanocomposites. Therefore, GO/ZIF-67 nanocomposites were used as a modifier on a screen-printed electrode (GO/ZIF-67/SPE) for studying the electrochemical behavior of epinine in phosphate buffer saline (PBS) at pH 7.0 with voltammetry techniques. The GO/ZIF-67/SPE showed greater electrocatalytic activities than the bare SPE. As a result, the GO/ZIF-67/SPE was utilized for additional electrochemical examinations. The epinine concentration determination was in the range 9.0 × 10^−8^ M to 5.0 × 10^−4^ M, and the limit of detection (LOD) as well as the limit of quantification (LOQ) equaled 2.0 and 6.6 nM, respectively. From the scan rate study, the oxidation of epinine was found to be diffusion-controlled, and the simultaneous detection of epinine and dobutamine were well achieved with the differential pulse voltammetric (DPV) technique. Moreover, the stability and reproducibility of epinine at the GO/ZIF-67/SPE was studied, and the use of the GO/ZIF-67/SPE to detect epinine and dobutamine in real samples was furthermore successfully demonstrated.

## 1. Introduction

Catecholamines play a significant role in the nervous system as central and peripheral neurotransmitters. These materials are generated by the sympathetic nervous system activation and function as neurotransmitters and hormones for monitoring the heart rate, brain muscles activities, blood pressure, glycogenolysis, body temperature, and fatty acid mobilization [1,2]. Therefore, researchers have concentered on the design of a simplified rapid technique for detecting catecholamines in bioscience, biotechnology, and also medicinal chemistry, in particular in neurochemistry [1,2,3].

Epinine, also known by the common names N-methyldopamine and deoxyepinephrine, is an organic compound and a natural catecholamine drug that is structurally related to the important neurotransmitters dopamine and epinephrine. Epinine has been found in plants, insects, and animals. Epinine is an active form of ibopamine, when after oral administration it is hydrolyzed by plasma esterase, which is currently being widely studied for the treatment of congestive heart failure [3,4,5]. One of the most prominent pharmacological characteristics of epinine is its ability to raise blood pressure, which was reported by Barger and Dale as early as 1910 [6]. In addition, epinine is suggested as a suitable substitute for epinephrine [7]. Although the use of epinine has many benefits, an overdose of epinine can be harmful to the human body and the use of this drug must be controlled in patients. Hence, epinine quantification in the samples of human serum and urine would be necessary to develop the life and drug sciences.

Another drug called dobutamine, 4-[2-[[3-(4-hydroxyphenyl)-1-methylpropyl]amino]ethyl]-1,2-benzenediol, has been introduced as one of the most important inotropic synthetic catecholamine medicines with heavy β-adrenergic activities. Dobutamine is commonly utilized for treating cardiogenic shock and heart failure [8,9,10,11]. In fact, dobutamine contribute importantly to functions of the central nervous, hormonal, renal, and cardio-vascular systems. Moreover, it increases the cardiac output or the heart rate that causes the improvement of left ventricular performance, lessens the central venous and pulmonary artery wedge pressure and diminishes the symptom of the congestive heart failure; nonetheless, the most dangerous side effect of dobutamine is increased risk of arrhythmia, including fatal arrhythmias [12].

Changes in the metabolism of catecholamines in the human body could result in some severe illnesses (hypertension, neuroblastoma, and pheochromocytoma). The proper detection of the lower or greater content of the body would allow recording the incidence of specific diseases at the early phase and prevent additional progression of the diseases. Moreover, the quantities of catecholamines in biological fluids possibly show their physiological contributions in the body [13,14,15]. Therefore, it is important to develop a highly selective, sensitive, precise, and cost-effective analytical procedure to measure epinine and dobutamine concentrations in biological fluids.

Many reports are mentioned in the literature for the determination of epinine or dobutamine using various analytical methods, including liquid chromatography, normal Raman spectroscopy, spectrophotometry, spectrofluorimetry, capillary electrophoresis, and chemiluminescence [16,17,18,19,20,21,22,23,24,25,26].

Although these methods have been successfully used, they are laborious and need complicated operation and costly instrumentation, which would restrict their utilizations. Moreover, experts in the field utilized electrochemical techniques because of benefits such as simplified operation, faster responses, very good reproducibility, acceptable stability, lower costs, and lower limits of detection (LODs) [27,28,29,30,31,32,33].

In addition, electrochemical determination with screen-printed electrodes (SPEs) has been a widespread utilization in clinical and biomedical areas for the detection of biological molecules (proteins/peptides, DNA, amino acids, and various metabolites) to diagnose or prognose diseases. These electrodes have easy modification techniques, including quickness, disposability, affordability, robustness, trace volume consumption, higher reproducibility, and decreased pretreatment requirements for samples, which would be encouraging to detect biological molecules in complex matrices [34].

Chemically modified electrodes improve mass transfer kinetics at low overpotential, resulting in the decrease of interferences’ effect and avoiding surface fouling [35,36,37,38,39,40,41].

Nowadays, various nanostructured materials have been developed and employed for electrochemical studies [42,43,44,45,46,47,48,49]. Nanotechnology reduces the sizes of nanoparticles (NPs) of raw materials and improves the functionality of physical properties of NPs [50]. Zeolitic imidazolate frameworks (ZIFs), an attractive subclass of metal–organic frameworks (MOFs), have gained increasing attention and have been used in many fields of science due to ultrahigh porosity, great surface areas, facility of synthesis, and accessible coordinative unsaturated sites compared with most of other MOFs [51,52]. However, these porous materials suffer from low electrical conductivity, electroactivity, and stability, which limits their use in electrochemical applications. Introducing high-conductive materials with remarkable mechanical strength such as graphene could be a solution to conquering this problem [53].

The present study attempted the synthesis and characterization of graphene oxide (GO)/ZIF-67 nanocomposites by using a simple synthesis approach. The prepared nanocomposites has been described by various techniques. The objective of the present research was designing and fabricating a SPE modified with GO/ZIF-67 nanocomposites as a novel electrode to detect epinine and dobutamine in aqueous buffer solutions and evaluating the analytical performance of this modified electrode by epinine quantification in the presence of dobutamine. At the end, we examined real samples to determine epinine and dobutamine using the proposed electrochemical sensor.

## 2. Experimental

### 2.1. Instruments and Reagents

According to the research design, we used the Autolab PGSTAT302N potentiostat/galvanostat monitored with the GPES software for electrochemical assessment and analysis. The SPE (DropSens, DRP-110, Asturias, Spain) contained 3 traditional electrodes, including a silver pseudo-reference, an unmodified or modified graphite working electrode, and a graphite counter. Moreover, we used a Metrohm 710 pH meter to measure pH.

Epinine, dobutamine, and each of the remained reagents were of analytical grade. Sigma-Aldrich has been chosen to supply these materials. In addition, orthophosphoric acid as well as the respective salts with pH values ranging between 2.0 and 9.0 were utilized to procure the buffer solution.

### 2.2. Preparation of GO/ZIF-67

GO/ZIF-67 was synthesized according to the literature. Fifty milligrams of GO was dispersed with a stirrer for 15 min, and then 20 mg of Co(NO_3_)_2_·4H_2_O were added to it. The mixture was stirred with a stirrer for one hour. Simultaneously, 50 mg of 2-methyl imidazole were dissolved in 20 mL of water. An imidazole solution was added to the initial mixture, and after stirring for one hour, the mixture was transferred to an autoclave and left at 100 °C for 3 h. Finally, the precipitate was collected after being centrifuged and washed three times with ethanol.

### 2.3. Preparing the Modified Electrode

The unmodified SPE was covered with GO/ZIF-67 nanocomposites in an aqueous solution (1 mL) and developed with the dispersion of 1 mg of the GO/ZIF-67 nanocomposites and 1 h ultrasonication. Then, 3 μL aliquots of a GO/ZIF-67 nanocomposites/H_2_O suspension solution were placed on the working electrode. After the solvent evaporated, the electrode surface was thoroughly rinsed with deionized water to wash away the unimmobilized modifier and dried at room temperature. The ZIF-67/SPE and the GO/SPE were prepared with the same method using ZIF-67 and GO, instead of GO/ZIF-67.

The surface areas of the GO/ZIF-67/SPE and the bare SPE were obtained by cyclic voltammetry (CV) using 1 mM K_3_Fe(CN)_6_ at different scan rates. Using the Randles–Sevcik equation for GO/ZIF-67/SPE, the electrode surface was found to be 0.095 cm^2^, which was about 3.0 times greater than that of the bare SPE.

### 2.4. Preparing the Real Samples

Dobutamine ampule (250 mg/ampoule; Exir Pharmaceutical Co., Tehran, Iran) was purchased, and the diluted solution was obtained by dilution with a 0.1 M PBS solution (pH equal to 7.0) and immediately utilized to determine dobutamine. Then, an appropriate content of the final solution was transferred to the electrochemical cell and consequently utilized to analyze dobutamine using the standard addition method.

Upon the samples collection, we used a refrigerator to store the urine samples and centrifuged 30 mL of the samples at 3000 rpm for 10 min. Then, we filtered the supernatant with a 0.45 μm filter. After that, 20 mL of the solution were transported into a 50 mL volumetric flask and then diluted to the mark with PBS at pH of 7.0. In the next stage, diverse contents of epinine and dobutamine were used to spike the diluted urine samples, and the epinine and dobutamine contents were analyzed by this new technique with the standard addition method for the prevention of further matrix effects.

## 3. Results and Discussion

### 3.1. Characterizing the GO/ZIF-67 Nanocomposites

#### 3.1.1. Energy-Dispersive X-ray Spectroscopy (EDX)

**Figure 1:**
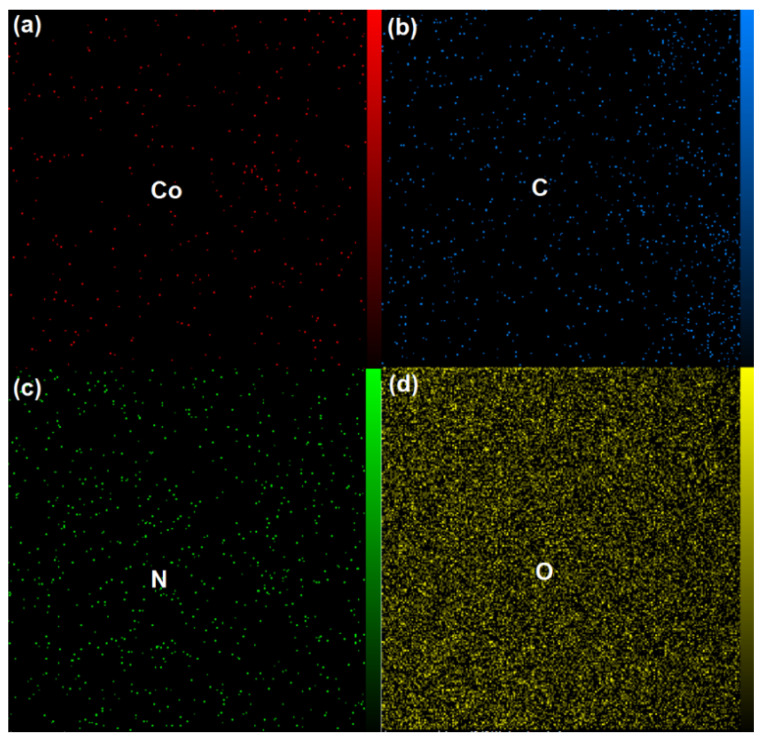

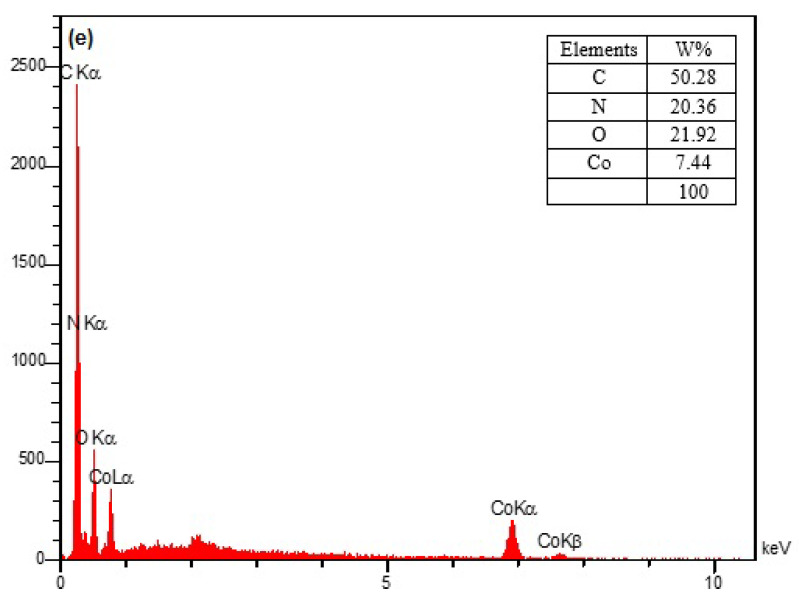
(**a**–**d**). Energy-Dispersive X-ray (EDX) mapping; (**e**) EDX spectra of nanoparticles in graphene oxide (GO)/ZIF-67 nanocomposites.

The results of Energy-Dispersive X-ray Spectroscopy (EDX) mapping analyses performed on GO/ZIF-67 nanocomposites are reported in Figure 1a–d. During the EDX measurement, different areas were focused, and the corresponding peaks are shown in Figure 1e. Both ZIF-67 and GO can be seen in the synthesized composite nanostructure in the EDX spectrum. These results confirmed the presence of Co along with C, O, and N. In addition, the results of EDX (Figure 1e) showed the weight percentages of the elements C, N, O, and Co were 50.28%, 20.36%, 21.92%, and 7.44%, respectively. In the EDX spectrum, the measured atomic % values were 1.7, 20.37, 58.66, and 19.20 for Co, N, C, O, respectively.

#### 3.1.2. FTIR Analysis

**Figure 2:**
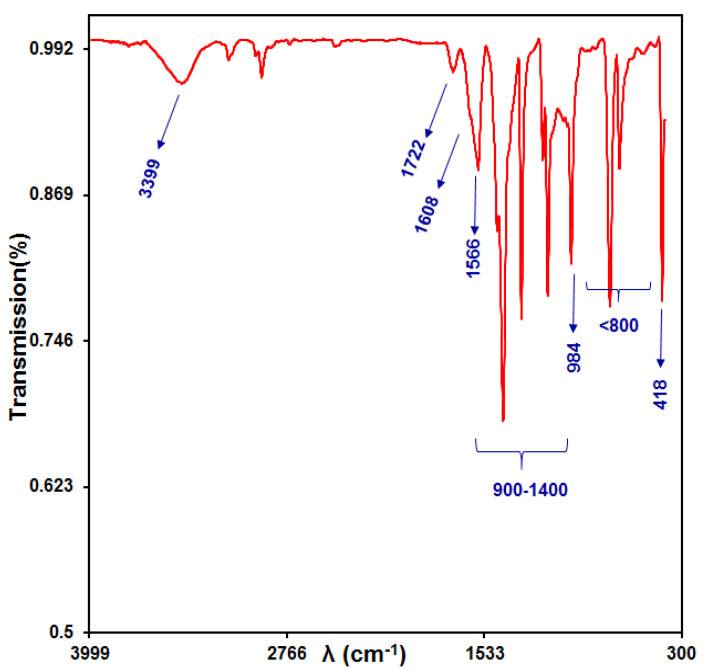
FTIR spectrum of the GO/ZIF-67 nanocomposites.

FTIR analysis was conducted with a Shimadzu 8400 spectrometer. In order to further demonstration the structure of NPs, the FTIR spectrum of the GO/ZIF-67 nanocomposites is presented in Figure 2. The FTIR peaks shown in this figure were exactly the same as those previously reported. Figure 2 shows all the peaks of 2-methyl imidazole. Only one extra peak was seen at 418 cm^−1^, which is the reason for the presence of the Co–N bond. The adsorption bands at 1722 and 984 cm^−1^ can be attributed to the C=O bending vibration and the C–N tensile in the GO/ZIF-67 nanocomposites, respectively. The peaks of ZIF-67 were shown at 1608, 1566, and 3399 cm^−1^. The related bands to C=C and C=N appeared at 1608 and 1566 cm^−1^, respectively. The widest band at 3399 cm^−1^ can be ascribed to the O–H stretching vibration in the solvent [54,55]. There were some sharp peaks in regions of 900–1400 cm^−1^ and below 800 cm^−1^ that may be assigned to the in-plane bending of the ring and out-of-plane bending, respectively [56].

#### 3.1.3. XRD Analysis

**Figure 3:**
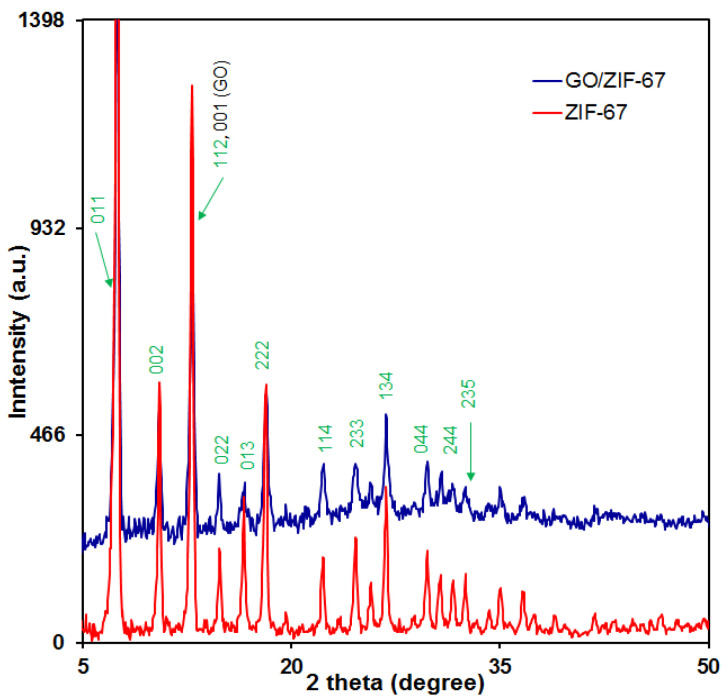
XRD patterns of the ZIF-67 nanoparticles and the GO/ZIF-67 nanocomposites.

XRD measurements were performed with X’Pert Pro (Panalytical, The Netherlands) at a scanning speed of 0.05° s^−1^. The structure information and the crystal phases of the synthesized ZIF-67 NPs and ZIF-67/GO nanocomposites by XRD patterns are indicated in Figure 3. As can be seen, there was a successful integration of ZIF-67 NPs into the GO/ZIF-67 nanocomposites. The indicative peak of GO at 11.96° appeared, but due to the overlap of ZIF-67 NP diffractions and other sharp peaks, this peak of GO was difficult to observe. The notable peaks of the GO/ZIF-67 nanocomposites in the XRD pattern were identified well with the ZIF-67 purity; these peaks can be mentioned as follows: 7.9° (011), 10.6° (002), 12.9° (112), 14.7° (022), 16.5° (013), 18.4° (222), 22.0° (114), 24.6° (233), 25.9° (002), 26.4° (134), 29.6° (044), 31.2° (244), 32.3° (235), and 43.0° (100).

#### 3.1.4. FESEM and TEM Spectroscopy

**Figure 4:**
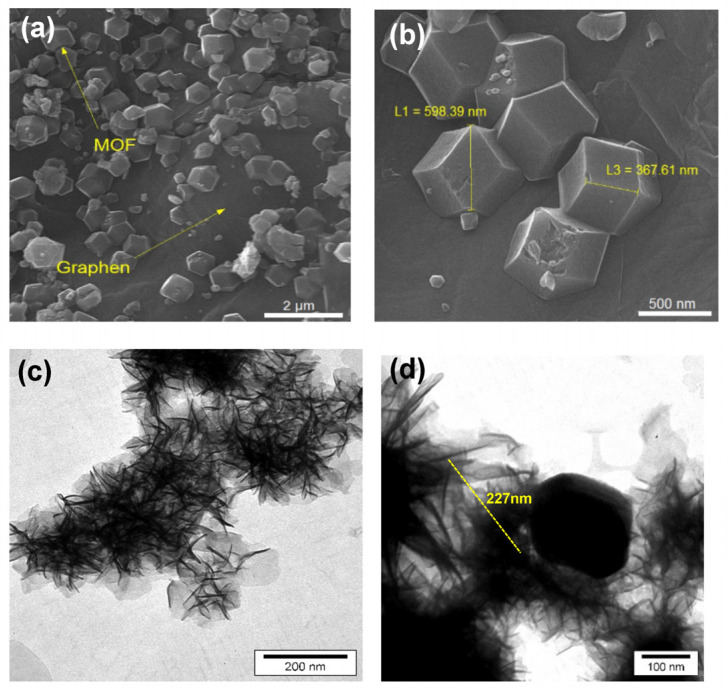
(**a**,**b**) FESEM image of the ZIF-67/GO nanoparticles at different magnifications. (**c**) TEM image of GO. (**d**) TEM image of the ZIF-67/GO nanocomposites.

The morphologies of the samples to study the grain size and the surface morphology were investigated by a field-emission scanning electron microscope (MIRA3TESCAN-XMU). Figure 4a,b shows the FESEM images of NPs at two different magnifications and indicates the presence of ZIF-67 crystals over the GO sheet. Figure 4a shows hexagonal ZIF-67 NPs scattered on the surface of GO. Figure 4b shows the sizes of the crystals of about 300 and 500 nm.

Figure 4c,d show TEM images of GO and GO/ZIF-67 nanocomposites by LEO912-AB instrument. The TEM analysis provided another evidence about the assembly of ZIF-67 over graphene sheets. Figure 4c displays that GO had many wrinkles and restacking in some areas on its sheet, and these phenomena were attributed to the existence of van der Waals forces.

#### 3.1.5. Electrochemical Impedance Spectroscopy (EIS) Studies

**Figure 5:**
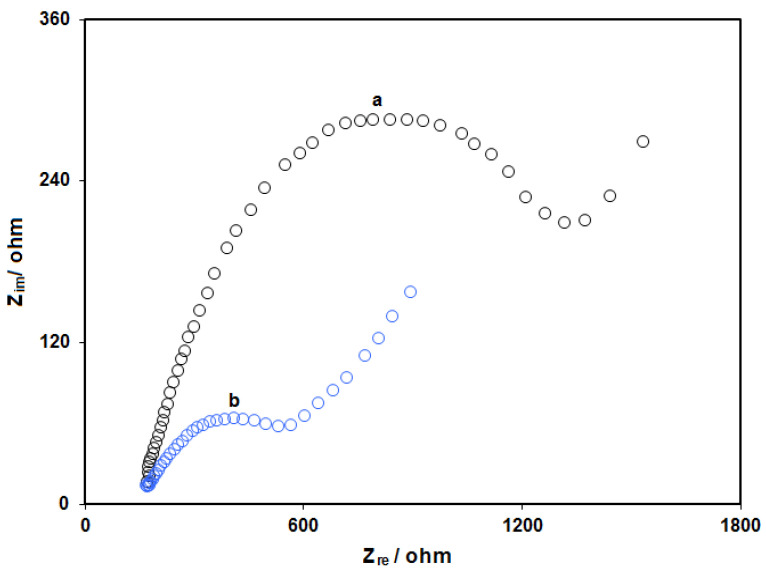
Nyquist diagrams at the bare screen-printed electrode (SPE) (curve a) and the GO/ZIF-67/SPE (curve b) in the presence of 0.5 mM [Fe(CN)6]^3–/4–^ at pH 7.0.

The modification of the electrode with ZIF-67/GO nanocomposites was tested by the EIS method in a solution containing 0.5 mM [Fe(CN)6]^3–/4–^ (Figure 5). As can be seen, the value of the charge transfer resistance at the surface of the bare electrode (curve a in Figure 5) was much higher than that of the modified electrode (curve b in Figure 5). This point confirmed the high conductivity of the ZIF-67/GO nanocomposites at the surface of the electrode.

### 3.2. pH Impact

**Figure 6:**
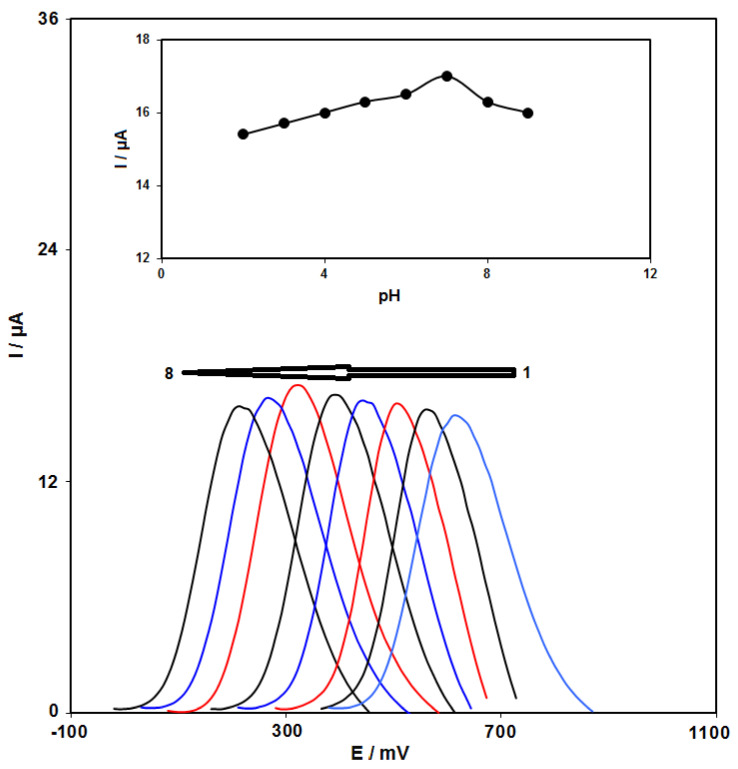
Differential pulse voltammetric graphs at the GO/ZIF-67/SPE in 0.1 M PBS with different pH values containing 200.0 μM of epinine. Numbers 1–8 correspond to pH values of 2.0, 3.0, 4.0, 5.0, 6.0, 7.0, 8.0, and 9.0. Inset: plot of Ip vs. pH.

In order to reach favorable peak current as well as suitable forms, we examined the pH impacts of accidental electrolytes on the anodic peak current and potential (Figure 6). Then, differential pulse voltammetry (DPV) was used to examined on numerous buffered solutions at pH in the range between 2.0 and 9.0 for the epinine solution. We observed that the most considerable peak current for oxidation at pH equal to 7.0, and thus, the reasonable pH value equaled 7.0 (the inset in Figure 6). Moreover, we selected 0.1 M PBS at pH 7.0 as the relevant electrolyte for the voltammetric analysis.

### 3.3. Electrochemical Behaviors of Epinine at the Surface of Various Electrodes

**Figure 7:**
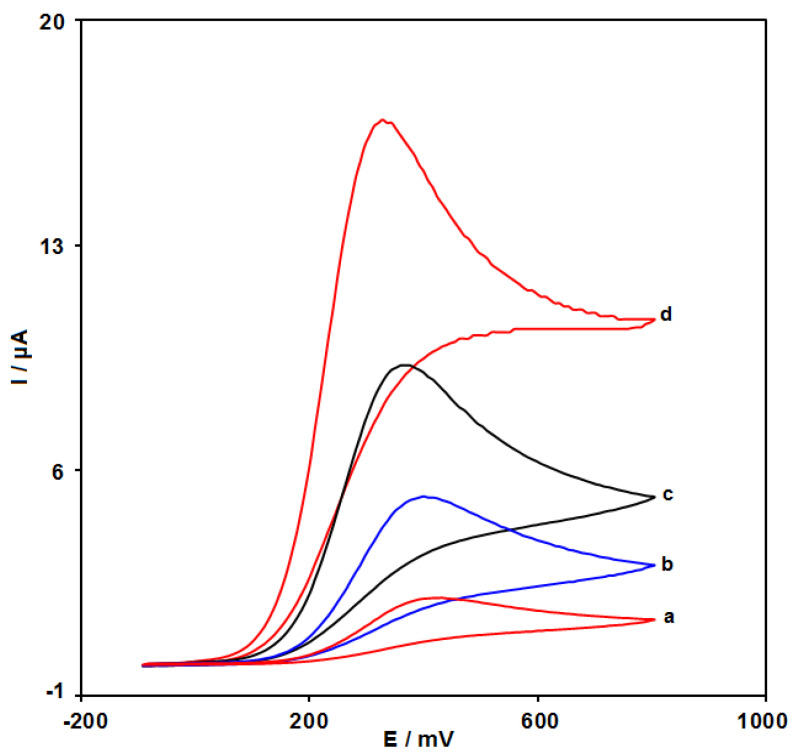
CVs at the unmodified SPE (curve a), the ZIF-67/SPE (curve b), the GO/SPE (curve c), and the GO/ZIF-67/SPE (curve d) in 0.1 M PBS at pH of 7.0 in the presence of 200 µM epinine at a 50 mVs^−1^ scan rate.

The electro-chemical behaviors of epinine at the unmodified SPE (curve a), the ZIF-67/SPE (curve b), and the GO/SPE (curve c), as well as the GO/ZIF-67/SPE, were studied using CV (Figure 7). The oxidation of epinine at the surface of the GO/ZIF-67/SPE occurred at a 330 mV potential, which would be nearly 40, 70, and 100 mV more negative as compared with the potentials at the GO/SPE, the ZIF-67/SPE, and the unmodified SPE, respectively. Moreover, the anodic peak current for epinine at the GO/ZIF-67/SPE was 8.5 times higher than the unmodified SPE currents.

It should be noted that the introduction of the GO/ZIF-67 nanocomposites enhanced the electrochemical catalytic activity of the bare SPE, which may be correlated to the greater surface area of the GO/ZIF-67 nanocomposites and the remarkable ability of the electron transfer of the nanomaterial.

### 3.4. The Impact of the Potential Scan Rate

**Figure 8:**
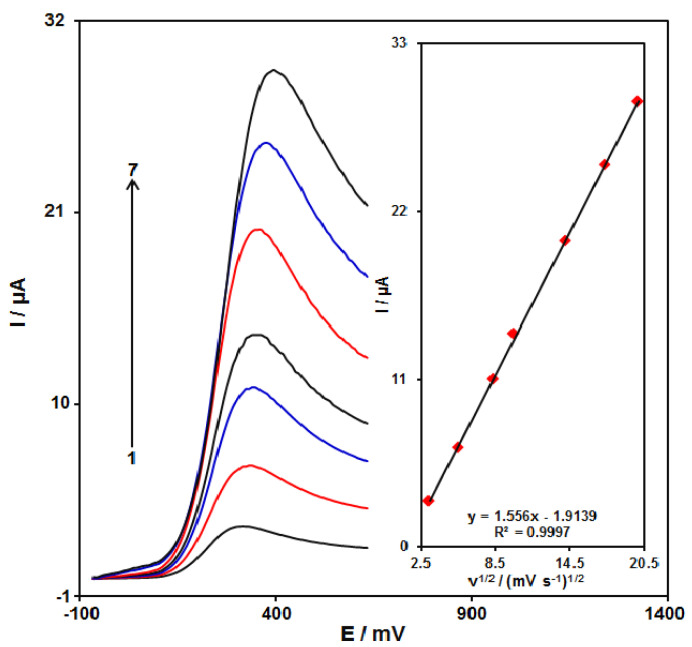
LSVs at the GO/ZIF-67/SPE in 0.1 M PBS at pH of 7.0 consisting of 100.0 µM epinine at different scan rates. Numbers 1–7 correspond to 10, 30, 70, 100, 200, 300, and 400 mV s^−1^. Inset: variations in the anodic peak currents vs. ν^1/2^.

The effectiveness of the oxidation peak current of epinine on the GO/ZIF-67/SPE was demonstrated by linear sweep voltammetry (LSV) at different scan rates. As seen in Figure 8, the peak current of epinine increased with the increasing scan rate. Moreover, a direct association existed between the current and the square root of the scan rate in a range of 10 to 400 mV s^−1^ (the inset in Figure 8), so that it could be a considerable association in conditions that epinine redox reaction was diffusion-controlled. As the charging current (i_c_) was dependent on v; hence, less scan rates were utilized for testing. However, testing would be lengthier at very lower scan rates. Thus, the 50 mV s^−1^ scan rate was selected as the optimum scan rate.

### 3.5. Tafel Analysis

**Figure 9:**
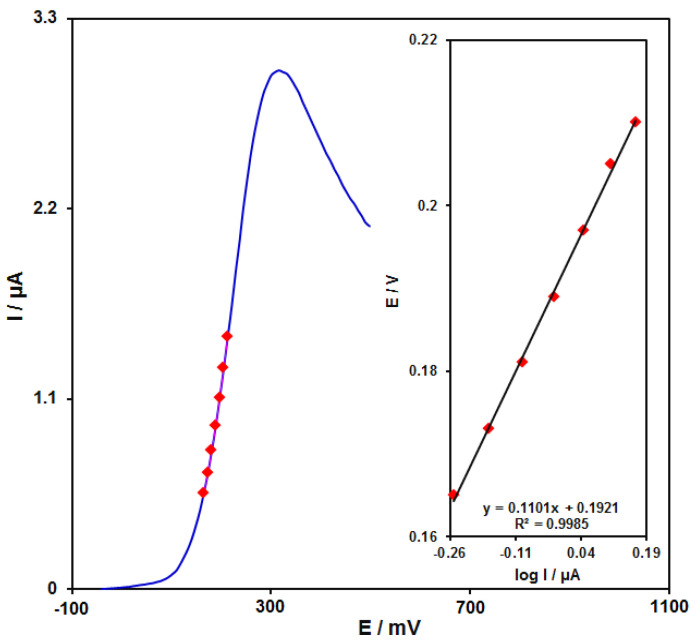
Linear sweep voltammograms at the GO/ZIF-67/SPE in 0.1 M PBS at pH equal to 7.0 with 100.0 µM epinine at a scan rate of 10 mV s^−1^. These points stand for the output utilized in the Tafel plot. As seen, the inset represnts the Tafel plot derived from the linear sweep voltammogram.

In this stage, a Tafel plot was drawn from the data obtained from the ascending part of the current–voltage curve recorded at a scan rate equal to 10 mV s^−1^ for epinine (Figure 9). It is notable that this piece of voltammogram, which was named the Tafel region, was influenced by the electron transfer kinetics between the substrate (epinine) and the GO/ZIF-67/SPE. Then, the Tafel slope of 0.1101 V was observed in complete agreement with the contribution of one electron in the rate determining the phase of the electrode [57], supposing the charge transfer coefficient α of 0.46 for epinine.

### 3.6. Chronoamperometric Examinations

**Figure 10:**
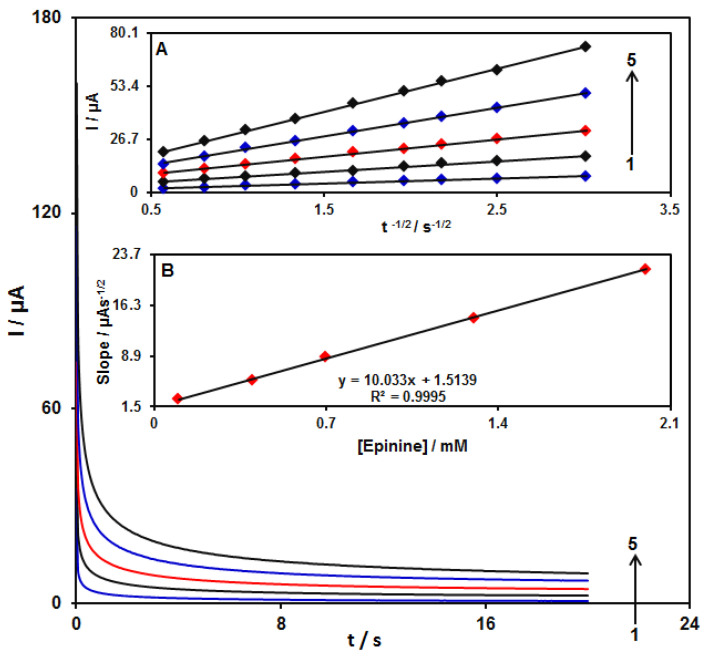
Chronoamperograms obtained at the GO/ZIF-67/SPE in 0.1 M PBS at pH of 7.0 for different concentrations of epinine. It is notable that numbers 1–5 correspond to 0.1, 0.4, 0.7, 1.3, and 2.0 mM of epinine. Inset (**A**) I versus t^−1/2^ observed by chronoamperograms 1 to 5. (**B**) Slope plot of the straight line vs. the concentration of epinine.

We performed chronoamperometric evaluations through the adjustment of the GO/ZIF-67/SPE potential at 430 mV as well as alterations in epinine concentrations in PBS (Figure 10). Then, we employed Cottrell’s Equation to illustrate current responses (I) for the diffusion-limited electrocatalytic procedure of electroactive materials such as epinine [57]:

*I = nFAD*^1/2^*C_b_π*^−1/2^*t*^−1/2^,

where D (cm^2^ s^−1^) represents the diffusion coefficient of the analyte; C_b_ represents the analyte bulk concentration (mol cm^−3^); F indicates the Faraday constant that equals 96,485 C; A stands for the geometric area of the electrode; n refers to the quantity of the electron exchanged in all reactant molecules. Upon drawing the I against t^−1/2^ plot, the linear curve was chosen from the raw chronoamperometric trace for different concentrations of epinine (Figure 10A). In the next stage, the slope of the final direct line was plotted as the opposed concentration of epinine (Figure 10B). Finally, the diffusion coefficient equaling to 8.6 × 10^−6^ cm^2^ s^−1^ was estimated for epinine, which was comparable with values obtained in references [27] (4.0 × 10^−6^ cm^2^ s^−1^) and [58] (6.8 × 10^−6^ cm^2^ s^−1^).

### 3.7. Determination of the Epinine Voltammetry

**Figure 11:**
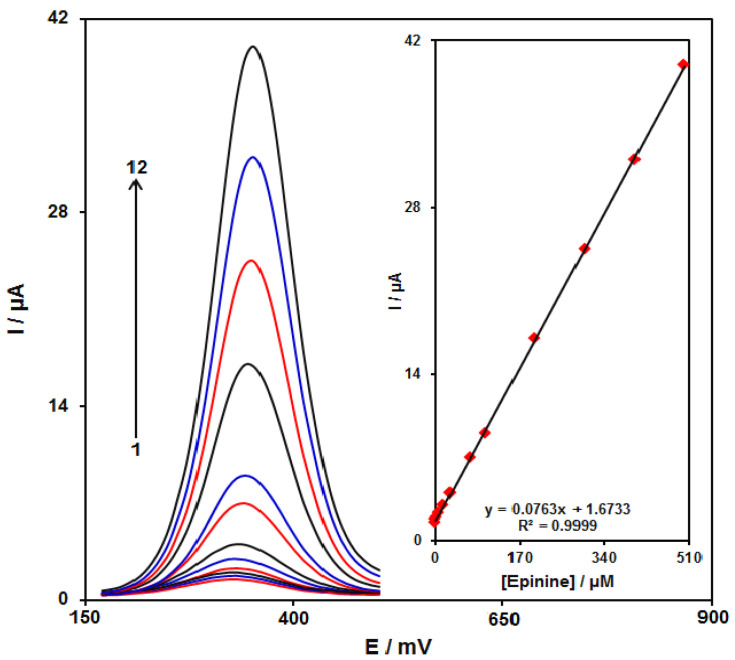
DPVs at the GO/ZIF-67/SPE in 0.1 M PBS (pH 7.0) containing different concentrations of epinine. Numbers 1–12 correspond to 0.09, 0.5, 2.5, 7,5, 15.0, 30.0, 70.0, 100.0, 200.0, 300.0, 400.0, and 500.0 μM of epinine. The inset shows the plot of the peak current as a function of the epinine concentration in the range of 0.09–500.0 μM.

At this stage, we measured the DPV based on the optimized conditions to achieve the epinine calibration plot. These optimum testing conditions for DPV measurements (i.e., modulation amplitude, 0.02505 V; step potential, 0.01 V; initial potential, 170 mV; and end potential, 500 mV) were used to generate the epinine calibration curve (Figure 11). Therefore, Figure 11 shows common DPVs for diverse concentrations of epinine. At the end, the slope value of epinine calibration curve of 0.0763 μA/μM was maintained in the epinine concentration range between 0.09 and 500 μM. Moreover, limit of detection (LOD) and limit of quantification (LOQ) were obtained 2.0 nM and 6.0 nM respectively (S/N = 3). The detection limit was comparable with the obtained values in references [27] (0.2 μM) and [58] (1.0 nM).

In addition, the optimum testing conditions for DPV measurements (i.e., modulation amplitude, 0.02505 V; step potential, 0.01 V; initial potential, 490 mV; end potential, 650 mV) were used to generate the dobutamine calibration curve. It has been also found that the dobutamine slope value equalled to 0.0697 μA/μM in the dobutamine concentration ranged between 0.4 and 1000.0 μM. Furthermore, the LOD of dobutamine equaled 0.1 μM (S/N = 3).

### 3.8. Simultaneous Detection of Epinine and Dobutamine

**Figure 12:**
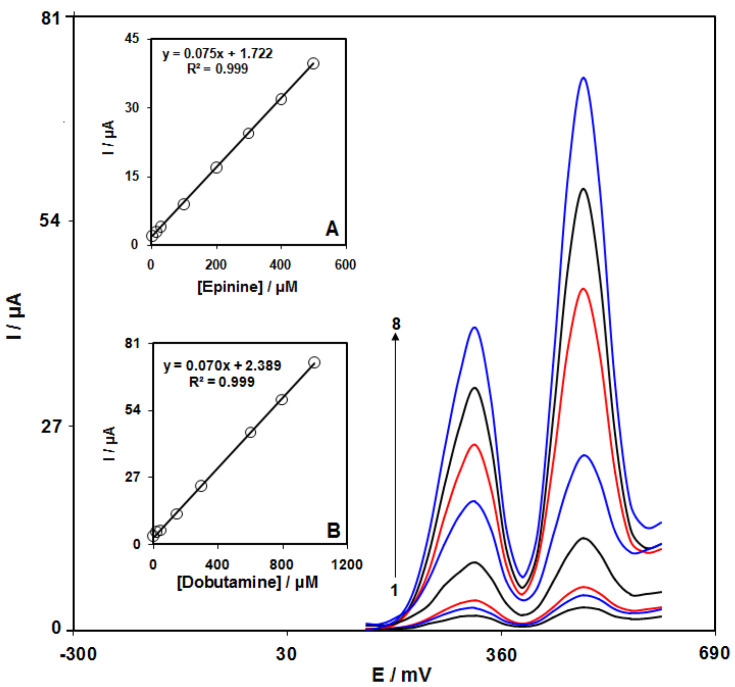
DPV graphs at the GO/ZIF-67/SPE in 0.1 M PBS at pH of 7.0 with various concentrations of epinine and dobutamine. Notably, numbers 1–8 correspond to 2.5 + 5.0, 15.0 + 25.0, 30.0 + 50.0, 100.0 + 150.0, 200.0 + 300.0, 300.0 + 600.0, 400.0 + 800.0, and 500.0 + 1000.0 µM of epinine and dobutamine. (**A**): Ip plot vs. epinine concentration. (**B**). Ip plot vs. dobutamine concentration.

The coexistence of epinine and dobutamine in several drug structures was confirmed; hence, it is necessary to simultaneously detect epinine and dobutamine. Therefore, we employed DPV for the simultaneous determination of epinine and dobutamine in the synthetic specimens using the GO/ZIF-67/SPE. Consequently, these two analytes were determined via a simultaneous enhancement of the concentration (Figure 12).

### 3.9. Stability and Repeatability of the Modified Electrodes

We evaluated the lengthy stability of the GO/ZIF-67/SPE for three weeks. Prior to the utilization of this modified electrode in the course of the mentioned time, we stored it at the atmosphere temperature and repeated the experiments. We did not observe any variation in the potential of the oxidation of epinine, but the current showed a reduction of <2.8% compared to with the 1st response. In the case of the epinine oxidization and the oxidization of the products, we recorded CVs to investigate the antifouling features of the modified electrode. Then, the cyclic voltammogram was registered around epinine after the potential cycling for 15 times at a scan rate of 50 mV s^−1^. Although these variations could not be seen in the peak potentials, a reduction of >2.6% was seen in currents. Therefore, using the modified electrode, the sensitivity as well as the pertinent oxidation product fouling influence decreased.

### 3.10. Interference Study

To evaluate the selectivity of the GO/ZIF-67/SPE, potential interfering substances were added to 50.0 μM epinine (PBS at pH = 7). The experimental results showed that there was no obvious interference for generally existing inorganic ions including K^+^, Cl^−^, Ca^2+^, Na^+^, Mg^2+^, NO_2_^−^, and some organic species such as vitamin B_9_, vitamin B_6_, tryptophan, alanine, citric acid, and phenylalanine. However, methyldopa, dopamine, levodopa, carbidopa, isoproterenol, and epinephrine with similar concentrations made interference in the determination of epinine. The data are presented in Table 1.

### 3.11. Analysis of the Real Samples

In this stage, we utilized the standard addition procedure in order to assess the usability of this new electrode for analyzing the spiked and real samples of epinine and dobutamine. Table 2 shows the experimental outputs, representing suitable recovery and reasonable relative standard deviation (RSD) values.

## 4. Conclusions

The present research reported the synthesis and description of the GO/ZIF-67 nanocomposite. The electrochemical response of epinine at the bare SPE and the GO/ZIF-67/SPE were studied by voltammetric techniques. The effect of pH on epinine showed the same numbers of protons and electrons in the electrochemical reactions, and the effect of the scan rate showed the diffusion-controlled procedure of the GO/ZIF-67/SPE towards epinine. The results showed that the GO/ZIF-67/SPE exhibited acceptable stability and reasonable repeatability and reproducibility toward epinine. The GO/ZIF-67/SPE proved to be effective sensors towards electrochemical investigations of epinine in the presence of dobutamine. The obtained outputs showed the GO/ZIF-67/SPE acted as a promising biosensor to determine biological molecules as well as devising the electroanalytical utilizations.

## Figures and Tables

**Table 1:** Study of the effects of some interferences in the determination of 50.0 μM epinine (n = 5). Each measurement was conducted with a new electrode surface.

Epinine Current (µA)	Interference	Percentage change in the Current in the Presence of Interference
5.5	K^+^, Cl^−^, Ca^2+^, Na^+^, Mg^2+^, and NO_2_^−^	±2%
5.5	Vitamin B_9_, vitamin B_6_, tryptophan, alanine, citric acid, and phenylalanine	3% to 4%
5.5	Methyldopa, dopamine, levodopa, carbidopa, isoproterenol, and epinephrine	90% to 100%

**Table 2:** Determination of epinine and dobutamine in real samples. All the concentrations are in μM (n = 5).

Sample	Spiked		Found		Recovery (%)		Relative Standard Deviation (RSD; %)	
	Epinine	Dobutamine	Epinine	Dobutamine	Epinine	Dobutamine	Epinine	Dobutamine
Dobutamine ampoule	0	0	-	2.5	-	-	-	3.2
	5.0	2.5	5.1	4.9	102.0	98.0	2.3	2.9
	10.0	5.0	9.9	7.7	99.0	102.7	3.5	1.9
	15.0	7.5	15.5	9.7	103.3	97.0	2.6	2.8
	20.0	10.0	19.5	12.7	97.5	101.6	1.8	2.4
Urine	0	0	-	-	-	-	-	-
	5.0	7.5	5.1	7.4	102.0	98.7	2.8	2.8
	7.5	12.5	7.3	12.6	97.3	100.8	3.5	1.9
	10.0	17.5	9.9	18.1	99.0	103.4	1.7	3.3
	12.5	22.5	12.6	22.2	100.8	98.7	2.7	2.4

## Data Availability

All the data are presented in the manuscript.
